# The Impact of Ethnicity on Wilms Tumor: Characteristics and Outcome of a South African Cohort

**DOI:** 10.1155/2015/706058

**Published:** 2015-03-26

**Authors:** D. K. Stones, G. P. Hadley, R. D. Wainwright, D. C. Stefan

**Affiliations:** ^1^Department of Paediatrics and Child Health, University of the Free State and Universitas Hospital, P.O. Box 339(G69), Bloemfontein 9301, South Africa; ^2^Department of Paediatric Surgery, Nelson Mandela School of Medicine, Private Bag 7, Congella 4013, South Africa; ^3^Paediatric Oncology Unit, Chris Hani Baragwanath Academic Hospital, Chris Hani Road, P.O. Box 2013, Bertsham, Soweto, Gauteng, South Africa; ^4^South African Medical Research Council, Francie Van Zyl, Parow, Tygerberg, Cape Town 7550, South Africa

## Abstract

*Background*. Nephroblastoma is the commonest renal tumour seen in children. It has a good prognosis in developed countries with survival rates estimated to be between 80% and 90%, while in Africa it remains low. *Method*. Retrospective study of patients diagnosed with nephroblastoma who are seen at 4 paediatric oncology units, representing 58.5% of all South African children with nephroblastoma and treated following SIOP protocol between January 2000 and December 2010. *Results*. A total of 416 patients were seen at the 4 units. Over 80% of our patients were African and almost 10% of mixed ethnicity. The most common stage was stage 4. The median survival was 28 months after diagnosis with the mixed ethnicity patients recording the longest duration (39 months) and the white patients had the shortest median survival. The overall 5-year survival rate was estimated to be 66%. Stage 2 patients did significantly better (85%). *Conclusions*. Our patients are similar with regard to gender ratio, median age, and age distribution as described in the literature, but in South Africa the more advanced stage disease seen than in other developed countries is translated into low overall survival rate.

## 1. Introduction

Worldwide over 80% of all childhood malignancies and 97% of the deaths related to childhood cancer occur in the developing world and with the poor resources and multiple other associated problems the survival rates of most childhood cancers do not approach the figures for the developed world [1]. Nephroblastoma or Wilms tumour is the most common renal tumour seen in children and accounts for 6-7% of cases of childhood cancer in the developed world [2]. In the South African Children's Tumour Registry they account for 12% of childhood cancers [3]. The overall survival rate of nephroblastoma approaches 90% in the developed world but in developing countries the survival rates are much less and in some sub-Saharan countries it is only 40% at 8 months after diagnosis [4, 5].

The tumour is supposed to be more common in Africa than in Europe and the USA with a median age at diagnosis of 42 [2]. The peak age is between 24 and 36 months and 75% of patients will be less than 60 months of age at diagnosis and 95% of patients will be less than 120 months of age at diagnosis. There is usually a slight female predominance [2, 4] but this is not seen in all studies [5–8].

There are two major treatment philosophies; there is the SIOP approach of neoadjuvant chemotherapy followed by surgery and continuation of chemotherapy depending on the stage at surgery while the National Wilms Tumor Study Group (NWTSG) approach is primary surgery followed by appropriate chemotherapy depending on the stage of the tumour. The primary goal of each is to treat patients with as little chemotherapy as possible and achieve the best possible survival rates. Both approaches achieve very comparable results.

There are very few studies from countries in Africa; one from Johannesburg showed the survival of white patients in South Africa as >90% while the black African patients did significantly worse with a survival rate of approximately 65% [9]. This study also showed that the stage 4 patients had a survival rate of <50% while stage 1 and 2 patients had rates that approximated 90%.

Two further studies showed a male to female ratio that varied from 1.85 to 1 to 1.3 to 1 [7, 8]. In Tanzania no stage 1 patients were seen and 80% of patients presented with advance disease [7]. The average age of the patients seen in a Dar Es Salaam study was almost 70 months, much older than the usual age for developed countries [7].

South Africa is a developing country with a diverse population and a discrepant health system.

It consists of a diverse population in terms of ethnicity but this can broadly be classified into black, white or Caucasian, coloured or mixed ethnicity, and Indians. The coloured population genetically consists of an ancestral mix between European and various Southern African black tribes. This ethnic group is widespread across South Africa but there is a higher concentration of this population group in specifically the Western Cape region. The Indians are predominantly found in Kwa Zulu Natal region situated in the eastern part of the country. Most of the patients are dependent on the public health services while there is a select group who can afford private medical care. This is an audit of the experience of four paediatric oncology units at different sites within South Africa. These units are located at public hospitals and treat predominantly indigent patients. Each of the units functions independently and all followed the SIOP principles including neoadjuvant chemotherapy. In South Africa all treatment is free of charge for children under 6 years of age and for older children the cost of treatment is directly related to their income. Transport and certain other costs are partially covered by nongovernmental organizations.

## 2. Methods

During the study period (2000–2010) 875 new patients with Wilms tumour were seen in South Africa. Of this number 165 were treated in centres following NWTSG principles and were excluded. Of the remaining 710 children 416 were managed in centres participating in this study and they form the study group. The other paediatric oncology units employing the SIOP protocol did not have complete data so they could not participate in this study. The 4 units that were elected to form part of the study represent a fair reflection of the population distribution in South Africa, Tygerberg Hospital, with larger coloured and white populations, while the other three units had a larger black population.

The 4 South African oncology units included in the study were the following.

- Inkosi Albert Luthuli Central hospital is a tertiary care public hospital in Durban which is allied to the Nelson R Mandela School of Medicine at the University of KwaZulu-Natal. The hospital provides oncology care to the population of KwaZulu-Natal, currently 10.5 million (4.2 million children), as well as parts of the Eastern Cape.
- The Universitas Hospital Academic Complex (UHAC) in Bloemfontein provides cancer care to over 1.5 million children. It is the referral pediatric oncology unit for the whole of the Free State and Northern Cape provinces, as well as some areas of both the North West and Eastern Cape provinces and Lesotho.
- Chris Hani Baragwanath Hospital is situated in Johannesburg and is the largest hospital in the world with approximately 3 200 beds. This unit serves the province of Gauteng as well as North West with occasional patients from Mpumalanga.
- Tygerberg Hospital Unit is situated in Cape Town in the Western Cape. Tygerberg Hospital is a tertiary hospital located in Parow, Cape Town, serving the Eastern Metropolitan region of Cape Town and the North-Eastern districts of the Western Cape province.

The diagnosis of nephroblastoma was made on the clinical examination and ultrasound and/or CT abdomen and in all cases that proceeded to surgery on the resected specimens. Before treatment patients had biopsies with pathological specimens which were processed and reviewed by the national health laboratory attached to the reporting institution.

The following information was extracted and further analysed: date of birth and age at diagnosis, sex and race of all patients, date of diagnosis, stage at presentation and the outcome, and date on which the patient was last seen or died. All patients were diagnosed between January 2000 and December 2010 and followed up till December 2011. There were 36 patient records in which there were certain elements missing from the data. All patients' gender, age, and race were documented. In 30 patients the stage of the disease was not documented while in 6 the outcome or date on which the patient was last seen was not documented. The data for each of the population groups was analysed separately. As the Asian group was so small the figures for this group were not further analysed but they were included in the analysis of the whole group of patients.

All results were analysed using Graphpad Prism 4 programme and survival curves calculated using the same programme.

Ethical approval was obtained by all 4 participating units and confidentiality of the patients was kept entirely during the study.

## 3. Results

A total of 416 patients with the diagnosis of nephroblastoma were seen in these 4 units over a 10-year period (Table 1). This comprises 58.5% of all nephroblastoma patients notified to the SA childhood cancer tumour registry.

There were 214 male and 202 female patients, with a male to female ratio of 1.05 to 1.

The median age of our patients was 42 months. The median age of the female patients was 43 months, 3 months older than the male cohort. The age range of our patients was from 0 to 178 months of age. Our peak age was between 24 and 36 months and 73% of our patients were <60 months at diagnosis: >95% of our patients were <120 months old at diagnosis. The median age of the mixed ethnicity group was the youngest at 31 months while the African group had a median age of 42 months. The white population had a median age of 40 months. Most of our patients, 356 (85%), were of African descent.

The stage of disease was documented in 386 patients. As all patients were treated on SIOP based protocols the staging was done after preoperative chemotherapy except for some of the stage 4 patients, who were staged at diagnosis, based on the imaging studies. The patients who were not staged included those who died soon after admission or failed to proceed to surgery and thus no formal staging could be performed. Stage 4 was the most common stage seen in the four units and comprised 112 (27%) of the patients; stage 1 and 3 patients each contributed 22% while stage 2 made up 15% of the patients. Our incidence of stage 5 was 8%. Of all our patients 50% had advanced stage disease at presentation or after the neoadjuvant chemotherapy.

**Figure 1:**
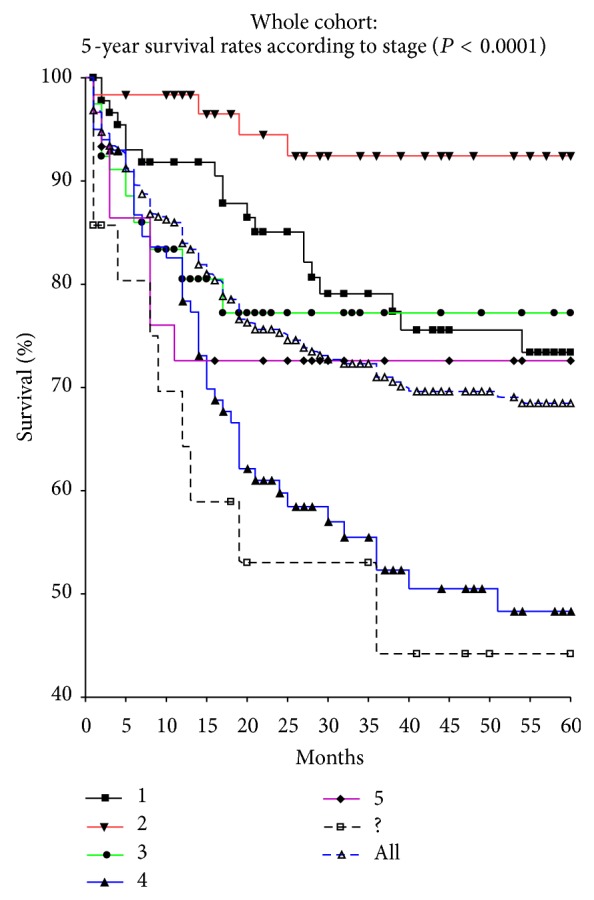
Whole cohort: 5-year survival according to stage.

The survival rates according to the stages showed, as expected, that stage 4 patients and those not staged did the worst. Stage 1 patients, which should do the best, had a survival rate similar to stage 3 patients while stage 2 patients did the best at 85% (Figure 1).

Amongst the African patients stage 4 was the most common (28%) followed by stage 3 at 22%. Stage 1 and 2 patients contributed to the remaining 34% of this group. In 8% of cases the patients had bilateral nephroblastoma. In the mixed ethnicity group 46% of the patients were stage 1 while stage 3 and 4 patients made up 17.5% each of the remainder. The incidence of stage 5 was 7.5% while stage 2 made up the remaining 10% of cases. In the white population stage 1 was the most common at 53% of cases; stage 4 comprised 28% and the remaining stages each made up 7%. The percentage of advanced disease was 50% in the African group while in the white and mixed ethnicity cohorts it was lower at 32% and 34%, respectively.

The outcome of 406 patients was assessed by documenting the date on which the patient was last seen or the date of death. In 10 patients the outcome or date on which the patient was last seen was not documented. The overall median survival rate was 27 months. A total of 281 patients were alive when last seen and their survival ranged from 0 months to 139 months with a median of 25-month survival after diagnosis. There were 130 patients who had confirmatory evidence of death; their survival ranged from 0 months to 132 months with a median survival of 28 months after diagnosis. The median survival rates for each of the ethnic groups were also analysed: the African patients had a median survival of 26 months compared to the mixed ethnicity group of 39 months while the white patients had a survival of 21 months. Our projected survival of all patients at 60 months after diagnosis was estimated to be 66%.

## 4. Discussion

This is the largest cohort of patients with nephroblastoma reported from South Africa and the largest from Africa [6–11]. Nephroblastoma is the commonest renal tumour that occurs in children and in developed countries it has an overall survival rate that approaches 90%. In developing countries, for a variety of reasons the survival rates seldom approach these figures [1, 10].

There were slightly more males than female patients in our study as it is described in the literature [2, 10] although other reports have shown that there is a varying male to female ratio that ranges from 2 : 3 to 1 : 1 [5, 6, 8].

The age range in patients with nephroblastoma is reported from under 3 months to 180 months of age [6, 10, 11]. Our youngest patient was less than 1 month of age while the oldest was 178 months of age which fits in with the age distribution as described in the literature. Other studies have reported different age ranges of patients that range from under 24 months to 180 months [6, 7, 10, 11].

The median age of children with nephroblastoma is reported to be 42 months of age [2, 8] which is the same as our median age of 42 months. There are reports of older median ages that approach 50–70 months [5, 7]. A study from South Africa showed a median age of 39 months and, although a somewhat different population group, it is very close to our median age of 42 months [6]. There is a study from the GFAOP group who report a median age of only 36 months in their cohort of patients [11].

The NWTSG studies report a median age for male of 36.5 months while females are somewhat older at 42.5 months [6]. Our females had a median age similar to the NWTSG study but our male cohort was about 3 months older. It is evident from this study that the median ages of the patients who died, whether they are female or male, are about 4-5 months older than those who survived.

The median age of the different racial groups was also very different with the black cohort being the eldest at 42 months and the mixed ethnicity cohort the youngest at 34 months: both very dissimilar to the studies from Cape Town and the NWTSG [6].

The peak age range for this study (between 24 and 60 months) was similar to previous reports, and more than 95% of our patients were diagnosed before 120 months of age.

The racial distribution of our patients reflects the population of South Africa which estimates that 80% of the population is black, 9% each of white and mixed ethnicity and 2.5% Asian origin [12]. Each unit has a different racial mix with two units seeing predominantly black patients, one mainly saw patients of mixed ethnicity while the last unit saw patients of all race groups. In another published study from South Africa [6], in an area of high prevalence of mixed ethnicity, the racial mix was 42% black patients compared to our figure of 85% while the mixed ethnicity was 45% compared to our 10% incidence. They also reported a higher proportion of white patients compared to our study (12% versus 3.6%). Our incidence of white patients was extremely low reflecting the locations of the units involved in the study and the communities they serve. These differing demographics impact the presentation and outcome of all patients, including those with Wilms tumour.

The study of the Groupe Franco Africain d'Oncologie Pediatrique (GFAOP) [11] of the treatment of nephroblastoma in 8 African pilot units showed an incidence of stage 1 disease of 35% while stage IV disease accounted only for 18% of their patients compared to our incidence of advance disease that approaches 50%. Their overall survival was 71.6%.

The SIOP figures from Europe also show a different stage incidence with almost 60% of cases being stage 1 after preoperative chemotherapy [4].

Figures from other African countries quote that over 70% of their patients had advanced stage disease at presentation; our figure, although lower, is still significant at just under 50% [5]. Our stage 4 patient ratios were very similar except for the mixed ethnicity patients who had the lowest incidence at 17%.

Our 5-year overall survival rate was 66% with a difference in the different stage survivals as well as a race difference with the white patients faring worst and the mixed ethnicity patients doing the best. The survival of the black patient was in between these values.

The survival rates for our patients according to stage showed that our stage 4 survival rate was lower than 50%.

Our overall survival shows that stage 2 patients fared better than stage 1 patients. This apparent paradox may be due to understaging of large tumours as stage 1 or due to the dominance of ethnic African patients in stage 2. Our stage 5 patients also appeared to do relatively well and had a survival rate of 63%.

*Limitations.* This is a retrospective study and limitations are related to the missing information or incomplete data.

Despite the fact that the histology was confirmed in all cases by a pathologist after surgery and all cases were discussed in a tumor board meeting further complete information about the pathological staging was difficult to obtain in all cases. This study is limited to the description of epidemiology, demographics, characteristics related to ethnicity, and overall survival of the South African patients.

No further information was collected regarding the nutritional status, socioeconomic implications, and associated morbidities which will be analysed and discussed in a future study.

## 5. Conclusions

This South African cohort is similar to reports from the developed world in terms of the gender ratio and age range. Our patients of mixed ethnicity were younger than our other two major population groups and we could not demonstrate the stage distribution reported by European groups using SIOP protocols where more than 60% of patients are stage 1 after neoadjuvant chemotherapy [13].

Our survival rate at just 66% after 5 years is significantly worse than that described in the literature for developed countries using SIOP protocol.

Our stage 4 survival is only 45%.

Awareness programs are essential as the diagnosis and treatment of early stages could increase the overall survival in South African children with nephroblastoma.

## Figures and Tables

**Table 1:** Characteristics of the South African cohort.

	Whole cohort	African	Mixed ethnicity	White	Asian
Total	416	356 (85.6%)	41 (9.8%)	15 (3.6%)	4 (1%)
Gender					
Male	214 (51%)	180 (50.5%)	24 (58.5%)	7 (46%)	3 (75%)
Female	202 (49%)	176 (49.5%)	17 (41.5%)	8 (54%)	1 (25%)
Age (months)					
Median	42	42	31	40	47
Range	0–178	0–178	3–167	18–149	51–157
Stages					
1	92 (22%)	65 (18.3%)	19 (46.3%)	8 (53.3%)	0
2	62 (15%)	55 (15.4%)	4 (9.8%)	1 (6.3%)	2 (50%)
3	88 (21.3%)	79 (22.2%)	7 (17.1%)	1 (6.3%)	1 (25%)
4	112 (27%)	101 (28.4%)	7 (17.1%)	4 (26.6%)	0
5	32 (7.7%)	27 (7.6%)	3 (7.3%)	1 (6.7%)	1 (25%)
Not staged	30 (7%)	29 (8.1%)	1 (2.4%)	0	0
Status					
Alive	281	238	29	11	3
Died	130	113	12	4	1
Unknown	5	5	0	0	0
Median survival (months)	27	26	39	21	53
